# Draft genome sequences of a *bla_CTX_*_-M-15_-harboring and an atypical enteropathogenic *Escherichia coli* isolate recovered from slaughterhouses in Nigeria

**DOI:** 10.1128/mra.00140-24

**Published:** 2024-06-11

**Authors:** Abeni Beshiru, Etinosa O. Igbinosa, Sascha Al Dahouk, Ralf Dieckmann, Szilvia Neuhaus

**Affiliations:** 1 Department of Biological Safety, German Federal Institute for Risk Assessment (BfR), Berlin, Germany; 2 Department of Microbiology, College of Natural and Applied Sciences, Western Delta University, Oghara, Nigeria; 3 Applied Microbial Processes & Environmental Health Research Group, Faculty of Life Sciences, University of Benin, Benin City, Nigeria; 4 Department of Environmental Hygiene, German Environment Agency (UBA), Berlin, Germany; Rochester Institute of Technology, Rochester, New York, USA

**Keywords:** slaughterhouses, genome sequencing, antimicrobial resistance, molecular epidemiology, public health, environmental health, bacteriology, microbial pathogenesis, food surfaces, food safety

## Abstract

We present the draft genome sequences of two *Escherichia coli* strains isolated from slaughterhouses in Edo State, Nigeria, in 2019. The isolates were identified as *bla_CTX-_*_M-15_-harboring (19-47-58) and atypical enteropathogenic *E. coli* (aEPEC) (19-47-66), belonging to multilocus sequence types (MLST) ST46 and ST2089, respectively.

## ANNOUNCEMENT

Extended-spectrum beta-lactamase (ESBL) producing *Escherichia coli* poses a significant public health concern, given the World Health Organization’s recognition of third-generation cephalosporins as critically important antimicrobials (1). Atypical enteropathogenic *E. coli* (aEPEC) represents a cause of severe gastrointestinal infections (2). Livestock can be carriers of both ESBL-*E. coli* and aEPEC (3, 4). Slaughterhouse environments may serve as an important contamination source of meat with potential pathogenic and antibiotic-resistant bacteria (5). *E. coli* isolates 19-47-58 and 19-47-66 were recovered from meat contact surfaces of Nigerian slaughterhouses. The isolates were recovered in 2020 with a swab and isolated on chromoccult coliform agar (Merck, Darmstadt, Germany) at 24 h/37°C. Genomic DNA was extracted from a single colony cultivated in lysogeny broth at 37°C for 24 h, using the PureLink Genomic DNA mini kit (Thermo Fisher Scientific, USA). Sequencing libraries were prepared with the Nextera DNA Flex Library Preparation kit (Illumina, San Diego, USA). Paired-end sequencing took place on the Illumina NextSeq 500 benchtop sequencer, employing the NextSeq 500/550 Mid Output Kit v2.5 (300-cycle) (Illumina, San Diego, USA) with 2 × 149 bp cycles. The DNA isolation and sequencing kits were used according to the instructions of the manufacturer. Default parameters were used for all software unless otherwise specified. Raw reads underwent trimming and *de novo* assembly through the AQUAMIS pipeline v1.3.11 (6). This pipeline utilized fastp v0.22.0 for trimming (7) and shovill v1.1.0 for genome assembly. In addition, the AQUAMIS pipeline incorporated mash v 2.3 for reference search (8) and quast v 5.0.2 for assembly quality control (9). Assembly characteristics are provided in Table 1.

**TABLE 1:** Summary of assembly characteristics of two *E. coli* isolates

Isolate ID	Size (bp)	High-quality reads^*a*^	G + Ccontent (%)	Contigs (n)	Q30 base fraction	N_50_	Read fraction majority genus	Coverage depth	Predicted genes (n)
19–47-58	4,506,737	2,377,277	50.8	266	0.813	86,720	0.916	76.9	4,309
19–47-66	4,940,629	3,218,373	50.6	310	0.916	84,609	0.992	96.4	4,339

^*a*^ Reads obtained after trimming (quality scores of ≥Q30).

The Bakcharak pipeline v3.0.4 (git version 1.0.0-240-g30246c8) (https://gitlab.com/bfr_bioinformatics/bakcharak) was applied for further analysis, including, among other tools, ABRicate v1.0.1 for AMR and virulence factor screening (https://github.com/tseemann/abricate), and mlst v2.23.0 for ST typing using the Achtman scheme (https://github.com/tseemann/mlst). Isolate 19-47-58 belonged to MLST ST46, serotype O8:H4, while isolate 19-47-66 belonged to ST2089, serotype O153var2/O8:H10. Notably, 19-47-66 featured the *eae* gene and was classified as aEPEC. Isolate 19-47-58 possessed 58 putative virulence genes, while 19-47-66 exhibited 117 putative virulence genes.

Both isolates showed similar resistance gene profiles, including *bla_EC_*, *bla_TEM-1_*, *aph*(6)*-Id*, *qnrS1*, and *tetA*. In addition, 19-47-58 carried *bla_CTX-M-15_*, *aph(3'')-Ib*, and *sul2*, while 19–47-66 carried *dfrA14* and *sul3*. Furthermore, both isolates showed mutations in *glpT*, whereas 19-47-66 displayed mutations in *gyrA*. For isolate 19-47-58, cephalosporine resistance was phenotypically verified using the MICRONAUT system with microtiter plate M/E1-247-100 for gram-negative bacteria (Merlin Diagnostika GmbH, Bornheim-Hersel, Germany). Results were interpreted based on European Committee on Antimicrobial Susceptibility Testing guidelines (10). This genomic information serves as a high-quality reference for future genome comparison studies. Ongoing investigations aim to discern the dissemination patterns of these isolates and their resistance determinants in both planktonic and biofilm states under diverse environmental stress conditions.

## Data Availability

The draft genome sequences of isolates 19-47-58 and 19-47-66 have been deposited in GenBank under accession numbers SAMN39716295 and SAMN39716296, respectively. The annotation of the draft genome file was added by the NCBI Prokaryotic Genome Annotation Pipeline (PGAP) during upload.
